# The Brazilian Center for Health Studies and the Brazilian Association of Collective Health: the issue of the nationalization of health in the Brazilian public health reform, 1976-1988

**DOI:** 10.1590/S0104-59702024000100030en

**Published:** 2024-07-15

**Authors:** Tiago Siqueira Reis

**Affiliations:** i Doctor of History, Universidade Federal Fluminense. Niterói – RJ – Brazil siqueira.treis@gmail.com

**Keywords:** Brazilian public health reform, Historiography of health, Brazilian Center for Health Studies (Cebes, Brazilian Association of Collective Health (Abrasco, Nationalization

## Abstract

An analysis is presented of the approaches taken by the Brazilian Center for Health Studies (Cebes) and the Brazilian Association of Collective Health (Abrasco) towards the nationalization of health during the Brazilian public health reform between 1976 (when Cebes was founded) and the enshrinement of public health in the Federal Constitution (1988). Discussions are presented of the theoretical and strategic principles defended by their intellectuals and the institutions’ positions towards the nationalization of health. By positioning themselves against complete nationalization, they did not break away from the privatizing rationale embedded in the prevailing model of healthcare, and endeavored to conciliate private interests within the new framework for public health.

The period of time stretching from the 1960s to the end of the 1980s witnessed intense political movements on a global scale, marked by the Cold War and the strong influence of the United States on countries from Latin America. As the struggle for global hegemony ensued, the countries from the Southern Cone experienced severe coups d’État that culminated in civil-military dictatorships. In Brazil, the dictatorship began in 1964 and lasted 21 years, significantly changing the population’s way of life and social struggles in the country (Dreifuss, 1981).

During the Brazilian dictatorship, the country’s health policy was marked, among other things, by the absence of a free, universal health system minimally equipped to serve the population. Instead, what prevailed were market forces, privatizations, geographical and social inequality of care throughout the country, inefficiency, and dependency on foreign manufacturers of medications and health equipment (Cebes, 1980). It was in this chaotic context that social struggles for improved health services began to gain force, galvanizing a diverse range of social actors inspired by progressive agendas calling for a comprehensive reform of the way public health was envisaged, delivered, and structured in the country, which became known as the Brazilian public health reform.

Most studies on the topic are underpinned by the idea that these collective agents can be identified as a single movement. The plurality of the organizations and agents working towards the public health reform is often mistaken for the worldviews of two entities: the Brazilian Center for Health Studies (Centro Brasileiro de Estudos de Saúde, Cebes) and the Brazilian Association of Collective Health (Associação Brasileira de Saúde Coletiva, Abrasco). Scholars from Cebes and Abrasco share the idea that the public health reform movement was institutional in nature, conceived with the creation of Cebes, in 1976, and subsequently consolidated with the founding, in 1979, of Abrasco. However, we suggest it would be erroneous to equate the creation of these two civil society organizations with the institutionalization of a broad-based, plural, and diverse movement of organizations and collective agents for the Brazilian public health reform (Reis, 2022).

This article investigates how Cebes and Abrasco – through the theoretical and strategic principles they espoused and the conceptions their intellectuals advocated – positioned themselves vis-à-vis the political action and theory developed around the nationalization of public health from the 1970s until 1988. To this end, we analyze the journal *Saúde em Debates* (Health in Debate), published by Cebes, as well as the documents produced by the 8th National Health Conference (Conferência Nacional de Saúde) and the contributions of the entities and their members in the debates about public health.

Our founding assumption is that these organizations did not fully break away from the privatizing and market-orientated rationale prevailing at the time in the health system in their efforts to lay new political and social foundations for a profound reform, since they supported conciliating private interests within the structural framework of public health. During the public health reform process, Cebes and Abrasco were in favor of retaining a role for the private sector in the reform project, arguing against the wholesale nationalization of health supported by other left-wing groups in the country that were engaged in the debate.

## Cebes and Abrasco

Cebes was founded on September 24, 1976, by members of the movements fighting for democracy and the transformation of healthcare in Brazil. For Escorel (1998), its creation was a turning point in the organization of the public health movement. Indeed, its early trajectory was marked by the political activism and higher education promoted by professors and students from the Department of Social and Preventive Medicine of the University of Campinas (Unicamp) as of the late 1960s. The majority of its intellectuals were inspired by Marxism, especially their reading of Gramsci (Fleury, 1989; Escorel, 1998), but also engaged with different thinkers from the social sciences in a bid to introduce a social historical perspective to the field of health.

Cebes organized its work along different axes: the production, communication, and distribution of the journal *Saúde em Debate*, participation in academic activities, and engagement in social and political actions that in some way touched on the area of health. The group claimed it was the theoretical and organizational pillar of the collective will of the country’s health professionals and civil society organizations that were fighting for the health sector and a return to democracy in the country, as Escorel (1998, p.88) points out:

Cebes ‘transposes’ a mode of thinking that emerged from the universities to the heart of civil society and coordinates the public health movement with the other social movements. It defends the interests of the population without being directly linked to it – in other words, in its trajectory, it was more directly linked to the academy or to institutional, parliamentary, or executive politics.

For Sônia Fleury (Sônia…, 2005), the idea that Cebes represented a “real public health party” also had to do with the perspective advocated by the Brazilian Communist Party (Partido Comunista Brasileiro, PCB), since party activists held a hegemony within Cebes; however, it also had members with different political leanings, making it impossible to say that Cebes was a branch of the PCB. Nonetheless, its policies were aligned with those of the PCB, such that “when everybody was keen to revolutionize the area of health, we envisaged making the reform because this was the position of the Communist Party” (Sônia..., 2005).

The journal published by Cebes, *Saúde em Debate*, translated and expounded its ideas, plans, and perspectives, becoming the main channel of communication for its interests and agendas. The first issue, launched in 1976, was followed by a further 19 until the end of 1987. Then in 1988, the year in which public health was enshrined in the new Brazilian constitution, a further four issues were brought out, as well as one special edition. The editorial line reflected the political agenda of the Cebes leadership and was geared towards academics and professionals from the area of health (Sophia, 2012).

Turning now to Abrasco, its creation was largely inspired by Cebes and it shared the same problems, ideals, and historical context. It was founded on September 27, 1979, during the first Meeting on the Development and Use of University-Qualified Personnel in the Area of Collective Health, held at the headquarters of the Pan-American Health Organization, in Brasilia. Composed of technical staff, professionals, professors, and students working in public health, the meeting aimed to “found an association that combined the interests of the different graduate level courses in that area” (Lima, Santana, 2006, p.19).

A Brazilian invention, the term Collective Health can be seen today in the political and academic agendas of countries from Latin America, the Caribbean, and Africa. Above all, it has to do with a way of addressing the relationships between knowledge, practice, and rights pertaining to quality of life. Instead of the traditional dichotomies – public health/medical care, curative medicine/preventive medicine, and even individual/society – the aim is to gain a new understanding whereby an interdisciplinary perspective and political debate around issues such as universality, equity, democracy, citizenship, and, more recently, subjectivity emerge as core issues. It was around these topics and the challenge of educating professionals attuned to the current of new ideas about health problems, some old, others the products of recent changes in the biomedical, political, and social fields, that in 1979 the Brazilian Association of Graduate Studies in Collective Health (Abrasco) was organized (Lima, Santana, 2006, p.9).

Abrasco’s membership included research and education entities and service providers in the area of collective health, and it aimed to support development in these areas, higher education, professional development, and public policymaking for health, education, science, and technology for the benefit of the population. Abrasco is essentially corporatist and academic in nature, but its range of actions is broad, enabling it to operate within Brazil’s social policies. To this end, as well as providing education for collective health professionals, it also worked alongside Cebes in important political endeavors as part of the public health reform movement (Belisário, 2002).

## Public health under the dictatorship, 1964-1985

During the dictatorship, public health was structured around social-security-based medical care. Health was basically organized by both the Ministry of Health and the Ministry of Social Welfare and Social Security. The former, with limited political and financial clout, had executive and normative functions and was focused on collective interests, as well as preventive medicine, disease surveillance, and vaccination programs. The latter was the recipient of virtually all the public resources and had great influence in the state apparatus. It was primarily responsible for individualized medical care, with a priority on curative and specialized medicine to the detriment of preventive and outpatient care or collective interests.

Social security was funded primarily through deductions on workers’ paychecks. Public social security funds were channeled into the acquisition of private healthcare services, resulting in the indiscriminate expansion of the private sector to the detriment of the public sector, as well as the acquisition of medical supplies from international companies, further exacerbating the dire foreign dependency of Brazilian public health. As a result, there burgeoned a giant industrial complex for capital accumulation from the provision of private medical care.

The public health project was spearheaded by the Ministry of Social Security, with the Ministry of Health taking a negligible role, resulting in a policy based on profiteering from the provision of services. Not only were public monies sunk into private hospitals through policies that prioritized hospitalization and individualized, curative medical care (Mello, 1976), but the health services were largely put in the hands of the private sector, enabling huge variations in the supply of services and availability of resources vis-a-vis the public sector. Public resources flowed into the private sector, undermining any policy that would strengthen establishments that were actually public. The private sector had a great many ways in which it rendered services: individual professional physicians working in clinics and offices; cooperatives and associations; hospitals; corporate health insurance etc.

This was the context of the privatization and commercialization of health in which a range of struggles for a public health reform took shape, including Cebes and Abrasco. Their agendas and strategies generally reflect the tendencies of the Brazilian left wing, since their members were active in its political parties.

These left-wing parties were strongly influenced by more general currents of ideas spreading throughout the international left-wing movements. Key amongst these was German social democracy, which was responsible for formulating the idea but it was no longer necessary to overtake and topple a state through a revolutionary process in order to attain the socialism envisaged by Marx, Engels, and Lenin. As the supporters of Eurocommunism suggested, the transition to socialism did not have to be revolutionary.^1^

Despite the different viewpoints of its interlocutors, the Eurocommunism transposed to the Brazilian context largely defended socialism through parliamentary democracy and as a peaceful way of beating capitalism. In the realm of health, it focused primarily on the public health reform, an example being the appropriation by Cebes and Abrasco of the idea of democracy as a universal value. Alongside the dissimilarities between the interlocutors of Eurocommunism and the differences in the ways its ideas were appropriated in different geographical areas, they all shared some core elements, which had a direct impact on the Brazilian left-wing debate within Cebes and Abrasco, namely:

1) socialism as a corollary of the installation of democracy, or democracy taken to its ‘extreme limit,’ to use the terms of Togliatti; 2) a conception of reform as a revolution diluted in time and thus having small, gentle impacts, with no violent ruptures; 3) a particular understanding of the state as an agent of transformations, depending on the correlation of forces among the classes, and thus capable of functioning to the benefit of workers; 4) a belief in suffrage as an effective means of vying for power, which may be obtained by the best organized player who has the inclination and power to accumulate the forces necessary for socialist construction; 5) an understanding that socialism would be achieved in stages, with the transition to socialism being preceded by an intermediate stage marked by the hegemonic presence of workers in state power structures. And there is also room for an addition: the Italian socialists, since Togliatti, have systematically rejected the perspective of the two-stage transition (a preliminary period of democratic struggle and soon afterwards a rupture) typical of the Marxist formulation of permanent revolution. This rejection was the very denial of the second act, the rupture (Dantas, 2017, p.56).

It is important to note that a great many members of Cebes and Abrasco were also members of the PCB in the early years of the public health reform, including figures such as Sônia Fleury, José Gomes Temporão, Jaime Oliveira, Sérgio Arouca, David Capistrano Filho, Hésio Cordeiro, Reinaldo Guimarães, and Carlos Nelson Coutinho. Despite the broader contextual influences and theoretical and political orientation exerted by left-wing parties through the members of theirs who belonged to Cebes and Abrasco, the public statements of these players and the articles in the specialized literature, most of which was written by these same individuals, would suggest the organizations were not manipulated and that their autonomy from political parties was prized (Jacobina, 2016).

If health was an inherently revolutionary field, then a combined effort was required to develop social theories for the area in order to guide the struggle. For Fleury (2018), the way to achieve this goal was to create and consolidate collective health as a research area geared towards a social policy and social theory of health. The convergence of social medicine with different disciplines from the social sciences enabled the social and political phenomena of health to be understood.

To a large extent, the intellectuals from Cebes and Abrasco wanted their organizations to be recognized as the objects and the political and institutional leaders in ensuring the hegemony of health through a public health reform. They were of the view that the composition of these organizations entitled them to “take the lead in institutional transformation but did not enable the necessary cultural change” (Fleury, 2018, p.55-56). As such, it was down to the public health movement, to wit, Cebes and Abrasco, to develop the theories for and take the lead in the political struggle in the field of health, which should encompass the whole of society.

## The issue of nationalization in the public health reform process

The editorial for the second issue of *Saúde em Debate*, published in 1977,^2^ contained a criticism of the changes the medical profession had undergone in the previous decades. Specifically, it pointed out the gradual disappearance of traditional medical practice; i.e., the independent contractor working from an office. It spoke out against state intervention in the provision of health services and the strong growth of medical businesses. This state of affairs trapped physicians in private business entities that even provided services under government contract, turning them into salaried workers. Yet in this capacity, they were not even entitled to the same rights assured to other categories of workers (Cebes, 1977a).

The Cebes editorial allowed that physicians be employed as salaried workers, but only if they earned salaries that were compatible with their status, had full labor rights, and provided services of utility to the population. The organization’s leadership understood that the delivery of health services needed reorganizing under a different perspective, breaking away from previous conceptions of the function of physicians and replacing them with new relationships between work teams as part of a broader struggle for a public health system.

As far as nationalization versus privatization was concerned, the editorial held that medical care for profit prevented the organization of adequate healthcare for the population. Salaried workers should engage in the struggle for “public, institutional, non-profit” healthcare whose “definition and orientation were set by entities that were legitimate representatives of service users” (Cebes, 1977a, p.3).

The same issue contained an article entitled “The privatization of government, philanthropic, university, and teaching hospitals.” Its author, the sanitarian Carlos Gentile de Mello, argued against the 1968 Coordination Plan for the Protection and Recovery of Health, also known as the National Health Plan, which proposed placing hospitals and other public administration healthcare facilities in the hands of the private sector. Mello (1977) warned of the perils of privatization, especially payment per unit of service, which was responsible for so many distortions and flaws in the health service.

The same topic was addressed in another article in the same edition of the journal. Its author, Regina Maria Gittoni, was at the time doing a master’s in political science at the University of São Paulo (USP) and would later become an important member of Abrasco. The article, “Privatize or nationalize?,” discusses the state of Brazilian public health and shows that the state could not be seen as a single entity. There were contradictory interests within the state apparatus involving disputes between the Ministries of Health and Social Security, dominated by different classes and class fractions. On the one hand there were those who supported big business, especially multinationals, whereas others defended small and medium-sized businesses. For Gittoni (1997), the state had been overrun by private-sector interests and agents who had clearly set their sights on healthcare. Therefore, it was important to understand these intrastate disputes, because nationalization concerned state action, whereas its actions in the context under analysis were under the control of business interests (Gittoni, 1977).

In the same year, the third issue of the journal had an editorial that claimed that the main barrier to public health reform was the “for-profit exploitation of health-related activities” (Cebes, 1977b, p.3). The Cebes leadership was against a public health policy that supported private-sector interests, although it did not explicitly state that public health should be left entirely in the hands of the public sector without any private involvement.

The issue repeatedly addressed by both the Cebes leadership and some of its most important members had to do with the democratization of health. Emerson Merhy wrote in the 4th issue of the journal that the problems of health would not be resolved through technical and rationalized schemes. This was because such a direction would be primarily political. Merhy argued that the state was dominated by different classes and fractions from the business sector operating in healthcare that denied the population’s interests. Democratization should be achieved not only by putting an end to the dictatorship, but also by allowing the working classes access to the state in the realm of health. If this did not happen, it would be impossible to democratize health (Merhy, 1977).

The Cebes membership and especially its different boards developed the idea that the relationship between state and health was rooted in the notion that the state was mistakenly identified with the dictatorship. In other words, nationalization was mistaken for control by the dictatorial government over the health sector. Given that the state apparatus was at that time riddled with different classes and fractions with business interests, any idea of nationalization would mean putting more power in the hands of these sectors while also centralizing the government’s authoritarian bureaucracy.

This perspective can be seen in the new Cebes plan of action for 1978 to 1979, which was approved in a national assembly of the Cebes membership. One of the main points, that “the state is therefore ‘nationalization in Brazil,’ has penetrated every sector of social life, not to bring about socialization (in the sense of greater social justice), but to enable a model of capitalist development” (Cebes, 1978, p.5).

It was in this specific context and the wider process towards redemocratization that the first Symposium on National Health Policy was held by the Health Committee of the Chamber of Deputies between October 9 and 11, 1979. Around 800 people took part from a range of civil society organizations, trade unions, and professional associations, as well as congresspersons of different persuasions (Teixeira, Jacobina, Souza, 1980). The proceedings were divided into three topics: human resources; hierarchy of health services; and privatization and nationalization of health services.

The event was a milestone in the discussions on public health in Brazil, not least because it took place on the invitation of the government in the midst of a dictatorship. The topic that interests us here is privatization versus nationalization, a debate that was kicked off with a speech by Paul Singer, a professor of economics from Unicamp. The members of the discussion panel represented different views, ranging from stronger to more nuanced advocates of both privatization and nationalization. For the purposes of the present discussion, the defense of privatization by the pro-business lobby is not of interest; rather, we shall home in on how nationalization was defended and in what respects.

For Singer (1980), the crux of the matter was not one of picking privatization or nationalization, but of discussing who would have control of health services. As he saw it, it was the consumer who should have this control. The main issue was how to empower service users directly so they could engage less asymmetrically with their physicians and other health workers. He was in favor of modifying the relationship between physician and patient by making medical knowledge accessible to users and informing them as to its limits.

The aim of providing users with some key basic knowledge was to render a change of stance on the part of physicians – whom he felt were hierarchical and monopolistic – making it less arbitrary. He saw it as indispensable for mechanisms of political control to be created by users at every level of the health service. Although he believed such solutions were closer to a nationalized service, he held that without making such alterations, “the big solutions, pertaining to state versus private” would change “the form more than the content of things” (Singer, 1980, p.162).

One of the key members of the panel was Guilherme Rodrigues da Silva, a professor from the USP Faculty of Medicine and a leading figure in Abrasco and Cebes. At the time, he was vice-president of the recently founded Abrasco, a position he held from 1979 to 1981. He also served as its president from 1987 to 1989. At the 8th National Health Conference, in 1986, he was the general rapporteur of the event.

In his analysis, the country was undergoing two movements at the same time: both nationalization and privatization. When the state took on responsibility for the health service, it directed its political and economic resources towards private healthcare providers. He felt that the nationalization versus privatization debate actually encompassed several other questions, including political choices, insofar as nationalization would not be enough of itself if the private sector continued to enjoy public patronage for the provision of public health services. Like Singer, he supported the complete nationalization of health services and understood that the biggest challenge was to create mechanisms to ensure workers had control of the services through authentic forms of representation (Brasil, 1980, p.165-168).

Another opinion worth commenting on is that expressed by Francisco Urbano de Araújo Filho, who was then secretary of the National Confederation of Agricultural Workers (Confederação Nacional dos Trabalhadores na Agricultura, Contag). Claiming that the entity he represented had the biggest contingent of health service users in the country at the time, he submitted an internal resolution in which he wrote that “there can be no doubt that the health service should be the direct responsibility of the state, directly responsible for the distribution of justice, especially social justice” (Brasil, 1980, p.178).

The Contag position, as expressed by its secretary, shows how impossible it was for there to be any plans for public health under the responsibility of private interests. Araújo Filho argued that however humanized and well-intentioned the private sector intended to be, it was inherently for-profit, making it incompatible with the goal of distributing health services to the whole population. Only the state could “act impersonally and impartially without considering the status of the client, seeing him as a human being, as a Brazilian, the same as every one of us, irrespective of economic, financial, intellectual, and other inequalities” (Brasil, 1980, p.178). Like Guilherme Rodrigues da Silva, Contag therefore understood that the state had been favoring the private sector and that only real nationalization could resolve the real issues of the health services.

This committee at the Chamber of Deputies was also a landmark in the trajectory of the public health reform when Sérgio Arouca, representing Cebes, read out the document “The Question of Democracy in Health” (Cebes, 1980), proposing for the first time the creation of the Unified Health System (Sistema Único de Saúde, SUS) (Paim, 2008). The article, penned by Hésio Cordeiro, José Luiz Fiori, and Reinaldo Guimarães, had been debated and approved by the members of Cebes. According to Paim (2008), it was a watershed in the development of the public health reform because this was when it ceased to be just an idea and became a concrete set of propositions (Paim, 2008).

The Cebes proposal encapsulated the primary claims of several groups engaged in the public health movement. It proposed social participation instead of authoritarianism; “instead of disease control policies, especially for transmittable diseases, the promotion of health and improved general quality of life; instead of a sector divided between public health and social security medicine, a universal, unified system” (Paiva, Teixeira, 2014, p.22).

For Cebes, the democratization of society and the socialization of politics would enable new channels to be opened up for the participation of a broad array of sectors of society. It would make the state apparatus receptive to the people’s interests and would offset unequal power relations. Centralized control would give way to the decentralization of power, including an institutional reform in health involving states and municipalities in the preparation of public policies that met their own particular needs. Another measure was based on “incorporating the legislation into all its levels as political representation of society in the state apparatus” (Cebes, 1985, p.11). Essentially, the plans for decentralization involved:

The whole authoritarian history of the country (an authoritarian history that culminated with the 1964 Regime, but which is part of the country’s republican history and tradition) often has a centralizing tendency, the tendency to concentrate power at the federal level; I believe that the construction and application of a public health reform should have a significant decentralized aspect, for the democratization and participation of all segments of the population. It must be highly decentralized, certainly taking as a basis the experiences of the Integrated Health Actions, and the reformulation and renewal of these experiences. And it should take account of the extreme heterogeneity of the organization of the health system in the different regions of the country, reverting the concentrationist model that has prioritized the south and southeast in the distribution of funding, facilities, and human resources. It means considering this heterogeneity; it perhaps means developing multiple unified health systems for states or regions under the orientation of one or more central bodies that develop and oversee a health policy, but whose execution, planning, and fine-tuning really happen in the specific context of the state and regional realities in order to overcome this concentration of power, this perverse and unequal model that discriminates so patently the many segments of the urban and rural population, north and northeast, south and southeast, dispersed populations and so forth (Cebes, 1985, p.11).

Sérgio Arouca’s interpretation of the document was that the health system should be decentralized and organized politically and administratively at each level (federal, state, and municipal). The aim of this decentralization was to

enable the authentic democratic participation of the population in the different levels and entities of the system, proposing and controlling the actions planned by its political organizations represented in government, assemblies, and entities from the unified health system itself. This is perhaps the key point of this proposal, which rules out a merely administrative or ‘nationalizing’ solution. It is a matter of channeling the claims and proposals of beneficiaries, transforming them into voices and votes at every level. In this way, a centralizing form of participation, so dear to the corporativist spirit and so susceptible to the coercive manipulations of a highly centralized and authoritarian state, as has traditionally been the Brazilian state, is also avoided. It establishes the terms of coexistence between salaried practice as part of the unified health system and the authentic practice of medicine in private offices, which are a tradition in Brazilian medicine (Brasil, 1980, p.229).

One factor in common in the Cebes editorials and the articles penned by its members is that there was no point just changing the plans for the national health system because the agents would retain the same business interests and the same privatizing and commercializing organization. The Cebes proposal that largely prevailed throughout the public health reform can be seen as a response to the problems raised by the aforementioned panelists debating privatization versus nationalization. The proposal would foster the democratization of health by decentralizing health services and creating mechanisms for its control by users. However, for democratization to leave the drawing board and become reality, it was not just enough for the government to simply change its political projects and plans for the national health system.

Cebes and Abrasco were unrelenting in their criticism of the centralization and interventionism inherent to the Brazilian dictatorial state. The organization of public health under authoritarian control was, they argued, complete “chaos.” They pointed out the existence of multiple modes of healthcare delivery and different forms of treatment and care, as well as inconsistencies in the fees charged (Cebes, 1985).

In the face of the chaos caused by the prevailing public health policy and the hegemony of the private sector, Cebes and Abrasco understood that there were “neither economic nor political nor technical conditions for a regime of democratic transition to leave out the private sector, blaming it simply for the problems of our healthcare” (Cebes, 1985, p.10). As a viable alternative to these problems, they proposed constructing a mediating, regulating state that would operate between private interests and the interests of the population, enabling diverse segments of society to engage in the development of public policies for health.

## Public versus private in the redemocratization process

In his article “Contributions for the definition of a healthcare policy for a government in democratic transition” published in the 17th issue of *Saúde em Debate*, Eleutério Rodriguez Neto (1985), formerly the president of Cebes, advocated a radical approach to the public health issue. Among other things, his proposal implied that the state should be responsible for the health system and the private sector should be given a complementary and subordinate role.

Decentralization was the linchpin of the proposal. For it not to be mistaken for a process of “statization” or “municipalization,” Rodriguez Neto (1985) explained that although decentralization should be contained in its aspects, it should actually extend beyond state and municipal boundaries to take shape as a “deconcentration of power amongst different levels until the level of the ‘frontline’” (p.15). This form of decentralization touched on all levels of administration in the system having decision-making power. However, it would remain multi-institutional, insofar as the federal government would participate “as a way of assuring equanimity of political, financial, and technical criteria in the development of this democratization process” (p.15).

Rodriguez Neto also argued that a new “compact” should be made between the public and private sectors, putting an end to the relationship between them that had prevailed under the dictatorship. This compact would involve: empowering the public sector as a model and standard of efficiency and efficacy; having service providers participate in discussions on payment for their services; regionalizing and hierarchizing private and public services in the same network; resizing the services contracted to take account of the real coverage needs and the priorities of the public sector in each region; and decentralizing quality control of care. In this new compact, the state should not subsidize or protect the private sector, leaving it in the hands of free enterprise (Rodriguez Neto, 1985, p.16).

Similarly, in a document published by the National Council of Health Secretaries (Conselho Nacional de Secretários de Saúde, Conass) entitled “The question of health in Brazil and guidelines for a program for a democratic government” it defended the establishment of a single health system that was decentralized and universal. As for the private sector, it stated that it should normatize and regulate its healthcare activities to ensure that the private sector “take an auxiliary role to that of the public sector in the Unified Health System, especially in the hospital network, where it has a particularly strong presence” (Conass, 1985, p.21-22).

Alongside these proposals, several others were proposed by different entities and congresspersons during the 5th National Health Policy Symposium, held at the Chamber of Deputies in November 1984. The final report provided guidelines for the transitional government, suggesting the continued involvement of the private sector in supplementing public health. It was in this context of transition and vying projects for Brazilian health that the 8th National Health Conference was held in Brasília in 1986.

This event was one of the key moments in the public health reform and is regarded in the specialized literature as crucial for defining its final format. It was also the first conference at which service users and organized civil society were allowed to take part in the debates.

One of the key sticking points during the conference was the idea of the complete nationalization of health versus a more gradual process of nationalization. The Workers’ Party (Partido dos Trabalhadores) and the Democratic Workers’ Party (Partido Democrático Trabalhista) were in favor of immediate nationalization, whereas the PCB and the Communist Party of Brazil, together with Cebes, Abrasco, and others stood for gradual nationalization through a strategy of conciliation with the private sector (Rodriguez Neto, 2019, p.91).

In his opening address, the president of the organizing committee, Sérgio Arouca (1987), said that even though the private sector was not at the event, it would be represented and its interests would be defended by those who were present. In other words, there should be no conflict with the private sector during the development of the public health reform. He also set forth as a principle for the discussions the idea that the public health project would not be drawn up without the involvement of the business sector. In stating this, he took the same line as Cebes and Abrasco insofar as he saw private interests as complementing the public health project rather than calling for the complete nationalization of healthcare. In his words:

A few days ago, some private sector entities withdrew from the Conference alleging that as they represented a high percentage of the health services provided in the Country, they should have more delegates. But they were wrong. As I understand it, this proportion of services does not correspond to the proportion of the Brazilian population. And this is a Conference for the Brazilian population and not a Conference of service providers. But I deeply regret their absence, because this Conference is addressing the creation of a national project that does not intend to exclude any of the groups involved in developing the health of the Brazilian people. As such, I wish to leave them this message: that even in their absence, we will be defending their interests, provided these are not the interests of the commercialization of health. Therefore, all businesspersons who are doing serious work in the area of health within their technical and professional sphere need not be afeared, because they will be defended here (Arouca, 1987, p.39).

Arouca’s position was followed closely by his colleagues throughout the conference. Jairnilson Paim questioned the extent to which the nationalization of the health service would meet the needs of the population. In his view, the opposite occurred when health was nationalized, since the organization of health would favor private interests. He wondered whether it was worthwhile maintaining support for the private sector, which he described as “incapable of maintaining itself in the market without the paternalistic protection of the State” (Paim, 1987, p.56).

Paim (1987) further questioned whether health services should be part of public authorities and whether health could be understood as a public service. The questions he raised are interesting, because he placed the difficulty of establishing democratic, universal healthcare that was accessible to all at the center of the debate. In his analysis, it was clear from the trajectory taken in Brazil that public did not mean state. Health policies and state services had over the years revealed themselves to be private, and not public, in nature. He indicated as an alternative the participation of citizens in health policies through the public control of state services and the management of institutions.

These views make it clear that one of the main issues the participants at the 8th National Health Conference tackled and which dogged the public health reform until the new constitution was passed in 1988 was whether the transformation of the health policy would involve its nationalization.

For the vice-president of Abrasco at the time, Sônia Fleury, the answer was “not necessarily”: it was not enough to nationalize health, making the state responsible for developing the national health policy, if a centralizing, authoritarian model that favored the private sector (as had historically been the case) was kept in place. She understood the state as being centralizing and authoritarian, especially since 1964, which was when the state apparatus and its administrative entities had undergone administrative and institutional modernization, making each entity more specialized and increasing the centralization and concentration of resources (Fleury, 1987, p.107).

At the 8th National Health Conference, Fleury (1987, p.120) argued that financial control by the state would be needed in the new social compact, but that this would not be enough, “making new legal definitions for this new relationship indispensable.” Hésio Cordeiro, president of Abrasco between 1983 and 1985, added that the relationship between the public and the private needed reformulating in order to avoid all manner of distortions, which meant it could not be achieved in the sphere of civil rights; in other words, the public health reform would require a new legal framework to govern the relationship between the public and the private, establishing a standard legal contract embedded in public law. As such, the private sector would provide services for the public sector based on a commitment to public and collective interests, established in a contract under public, not civil, law (Cordeiro, 1987, p.148).

There are already some experiments in Brazilian society in which essential services, such as transportation and telecommunications, are state monopolies, with the services being provided by private companies under concession. In this case, the service is assumed to be an essential public good, allowing the State legal mechanisms of control and intervention over the private service providers. The applicability of these experiments to the health sector must be assessed (Fleury, 1987, p.110).

The public-private relationship envisaged by Fleury could be achieved if there were a democratic state capable of socializing the policy, enabling effective decentralized and deconcentrated social control. As she saw it, nationalization was linked directly to a state technical bureaucracy that would underwrite capital accumulation.

Along similar lines, Cristina Possas, who was at the time a researcher with Fiocruz and a member of Cebes and Abrasco, argued for a reformulation of the legal and institutional relationship between the public and private sectors. She argued for the decentralization of health, to be achieved, among other things, through private sector concessions, which would compliment the health system. For Possas (1987, p.247), the concession model then adopted in other sectors, such as telecommunications and transportation, “would lend the State greater control and potential for intervention in the procurement of private health services.”

This would occur concomitantly with the creation of entities for the participation of civil society and public health system workers. Taken together, these factors would be capable of “overcoming the current distortions in the relationship with the private sector, assuring its subordination to public interests” (Possas, 1987, p.247). It was not, she felt, a matter of “going back over the timeworn debate of nationalization versus privatization of the health system, but of assuring new forms of relationship between the public and private sectors at the lowest social cost, marked by transparency and subject to democratic planning” (p.250).

In the conception of Eleutério Rodriguez Neto, the proposed reformulation of the health service and creation of a unified health system was not designed to nationalize health or eject private initiative from it:

The standards prevailing in the necessary relationship between the public authorities and the private sector should be subordinated to the technical and financial requirements for universal and equitable healthcare coverage, whose conditions may or may not be accepted by the contracting party for their participation or not in the public system of healthcare services. We are not talking about free enterprise financed directly by private, individual, or cooperative resources (insurance), which, provided it does not violate ethical standards, may be organized freely and independently of state control (Rodriguez Neto, 1987, p.263).

Along similar lines, at the same event the National Confederation of Workers (Confederação Nacional das Classes Trabalhadoras), represented by Luís Roberto de Oliveira, argued in favor of the creation of SUS, to be under the exclusive control and responsibility of the State. It suggested that the health system should “prioritize primarily the public sector and the non-profit private sector, represented by the Santas Casas [establishments run by the Sisters of Mercy] and charity hospitals, and that the for-profit sector should have a supplementary role and be subordinated to official control, the control of the state” (Brasil, 1987, p. 229).

However, at the 8th National Health Conference, the representative of the Unified Workers’ Central (Central Única dos Trabalhadores, CUT), Arlindo Chinaglia Júnior, rebutted Fleury’s arguments, pointing out that the debate over health could not ignore the power relations within society. In the name of CUT, he stated that society and health could only really be transformed if they were put in the hands of the workers, “but this is not on the agenda at the moment. At the moment, in fact, what there is is a discourse and, in practice, an alliance of classes, including with the dominant class” (Brasil, 1987, p.120). He also added the moot point that “under the aegis of the fact that nationalization may be authoritarian, we may witness the door being opened quite blatantly to private initiative, irrespective of the discourse” (p.120).

CUT’s response to Fleury’s was that they were in favor of the nationalization of health provided it was under workers’ control so as to break away from authoritarianism. The presentation ended with a call for the participants to defend nationalization, arguing that this strategy could not be placed on the negotiating table at state level, since the strategy should be guided by the organization, awareness-raising, and struggle of the working class (Brasil, 1987, p.120).

Finally, in the presentation of the pre-conference meetings held at state level, only the state of Goiás presented an alternative proposal to the auxiliary participation of the private sector in the public health system. This state’s representatives were in favor of the “gradual nationalization of the hospital and outpatient network, responsible for providing services for the population, and identified temporarily private institutions as having functions supplementary to the system” (Brasil, 1987, p.358).

## Final considerations

Throughout the public health reform process, until 1988, the strategy employed by Cebes and Abrasco was to win rights for citizens in the area of health by taking their grievances inside the state apparatus. For Dantas (2020), if the claims for democratization as a universal value, based (amongst other things) on a return to electoral politics, along with the democratic control of the state, the socialization of politics, and consequently the organization of a progressive civil society, uniting different classes and class fractions around a plan for public health, “did not produce an underestimation of the forces representing capital, at least it relativized its class role and overestimated the actual power of a left-wing struggle that was growing and was deliberately channeled into the state apparatus” (p.168).

In his analysis, alongside the arguments put forward by the public health movement (especially by Cebes and Abrasco) against the nationalization of health, the motivations seemed to have been different, underpinned by their strategies of

multiclass convergence in the defense of democracy – a democracy assumed by the majority left-wing forces at the time as a ‘universal value,’ namely, a frontier that should not be crossed, a limit of respect for the order and the rules of the democratic game that was held to express a commitment among classes not for a coup (bourgeoisie) but for a peaceful route towards socialism (workers) (Dantas, 2020, p.169).

Certainly, Cebes and Abrasco’s democratizing strategy addresses most of the arguments against the nationalization of Brazilian health. The conciliation among classes resulted in the private sector not being identified as a class enemy in the political project shared by these two organizations. The democratization of health was placed above classes; yet they may have overestimated themselves more than they underestimated the forces of capital. The private sector was not held up as a class enemy, as incompatible with the public health reform; the plans were based less on staving off the enemy and more on the purpose; i.e., democratic and universal healthcare.

It was felt that health issues would be resolved by the existence of a public system capable of providing universal, equitable, democratic health care for all. In other words, the state should provide and promote public health for the whole population, but would not meddle in the private sector and in free enterprise in the area of health. Rather, it would alter the relationship between the public and the private in the form it had taken under the dictatorship, when considerable resources and power had been diverted to the private sector. In this sense, ultimately the reform was based on the idea that every actor was entitled to their share of the area of health in a state of free competition. Yet rather than there being two different and opposing types of healthcare, they proposed and advocated a private sector that engaged with the public sector through direct public contracts and agreements, supplementing the strictly public services.

The argument that the total nationalization of health was hampered by the broader context because the private sector enjoyed significant – arguably hegemonic – power in the sphere of health resulted in a strategy based on the “dialectic of the possible.” If complete nationalization was not possible, then the private sector should be left with its free enterprise and the public sector with its public and universal healthcare. However, this was not what Cebes and Abrasco jointly proposed, because they believed that the new model for health that would be enshrined in the new Federal Constitution could not evade the involvement of the private sector in the provision of public services due to the deficiencies of the existing resources (physical, material, care etc.).

As a result, the public health system was born with the private sector grafted to its institutional structure. For the groups, the private sector was not the problem in the public health reform plans; the problem was the way the public sector related to the private sector. The space yielded to business interests in the public health project was not the outcome of clashes with the private sector, but of a decision to avoid conflict and to prioritize harmony between opposites. Cebes and Abrasco ultimately defended the private sector even though they were not its representatives.

## NOTES

1 Broadly speaking, Eurocommunism refers to the communist parties – especially in Italy, France, and Spain – that took a different perspective on communism than the USSR and proposed alternatives for international socialism based on a Western European standpoint and the defense of democratic socialism.

2 The members of the journal’s editorial board were: Ana Maria Segall Corrêa, Dalmo Herrera Feitoza, David Capistrano Filho, Emerson Elias Merhy, José Ruben F. Alcântara, and Sandra Roncali Mafezolli.
