# Supplementary material for: The Brazilian Center for Health Studies and the Brazilian Association of Collective Health: the issue of the nationalization of health in the Brazilian public health reform, 1976-1988
Source: Hist Cienc Saude Manguinhos. 2024 Jul 15;31:e2024030. doi: 10.1590/S0104-59702024000100030en (PMC11249365; doi:10.1590/S0104-59702024000100030en)
Supplement: Translation

# Translation: O Centro Brasileiro de Estudos de Saúde e a Associação Brasileira de Saúde Coletiva: a questão da estatização da saúde na reforma sanitária brasileira, 1976-1988

**DOI:** 10.1590/S0104-59702024000100030

**Authors:** Tiago Siqueira Reis

**Affiliations:** i Doutor em História, Universidade Federal Fluminense. Niterói – RJ – Brasil siqueira.treis@gmail.com

**Keywords:** Reforma sanitária brasileira, Historiografia da saúde, Centro Brasileiro de Estudos de Saúde (Cebes, Associação Brasileira de Saúde Coletiva (Abrasco, Estatização

## Abstract

Analisa como o Centro Brasileiro de Estudos de Saúde (Cebes) e a Associação Brasileira de Saúde Coletiva se comportaram em relação à questão da estatização da saúde pública no processo da reforma sanitária brasileira entre 1976, ano de criação do Cebes, até a institucionalização da saúde na Constituição Federal em 1988. Discutem-se os princípios teóricos e estratégicos defendidos por seus intelectuais, bem como os posicionamentos institucionais das agremiações ao tema da estatização da saúde. Partimos da hipótese de que, ao se posicionar contrárias à estatização integral, não romperam com a lógica privatizante do modelo de saúde até então vigente, uma vez que tentaram conciliar o privado no arcabouço estrutural da saúde pública.

Os anos 1960 até o final da década de 1980 foram de intensa movimentação política a nível global, marcados pela Guerra Fria e pela forte influência dos EUA nos países da América Latina. Nesse contexto de luta por hegemonia global, os países do Cone Sul sofreram duros golpes de Estado, culminando em ditaduras de natureza civil-militar. No Brasil, a ditadura iniciou em 1964, prolongando-se por longos 21 anos, alterando significativamente o modo de vida da população e das lutas sociais (Dreifuss, 1981).

Durante a ditadura brasileira a política nacional de saúde pode ser caracterizada, entre outros fatores, pela ausência de um sistema digno de serviços à população, gratuito e universal, prevalecendo mercantilização, privatização, tratamento geográfico e socialmente desiguais aos usuários em todo o território nacional, ineficiência e dependência externa na produção de medicamentos e equipamentos de saúde (Cebes, 1980). Diante do quadro caótico da saúde pública, intensificavam-se as lutas sociais pela saúde na sociedade civil a partir de uma diversificada composição de atores sociais orientados por agendas progressistas a favor de uma ampla reforma na maneira de conceber, praticar e estruturar a saúde pública no país, nomeada reforma sanitária brasileira (RSB).

Em grande parte dos estudos sobre o tema domina a ideia de que tais agentes coletivos são identificados como sendo o “movimento sanitário”. A pluralidade de agentes e organizações coletivas em prol da reforma sanitária é muitas das vezes confundida como sendo representante das visões de mundo do Centro Brasileiro de Estudos de Saúde (Cebes) e da Associação Brasileira de Saúde Coletiva (Abrasco). É consenso entre os intelectuais do Cebes e da Abrasco a ideia de que o movimento sanitário possui uma institucionalidade concebida a partir da criação do Cebes, em 1976, e posteriormente aglutinada pela fundação da Abrasco, em 1979. Por outro lado, chamamos atenção para o fato de não confundirmos a criação do Cebes e da Abrasco como organizações coletivas da sociedade civil com a institucionalização de um movimento amplo, plural e diverso de organizações e agentes coletivos em luta pela RSB (Reis, 2022).

Este artigo analisa como Cebes e Abrasco, a partir de seus princípios teóricos e estratégicos, bem como das concepções de seus intelectuais, comportaram-se no que diz respeito à teoria e à ação políticas relativas à questão da estatização da saúde pública entre 1970 e 1988. Para tanto, analisamos a revista *Saúde em Debates*, editada pelo Cebes, bem como a documentação referente à oitava Conferência Nacional de Saúde (CNS) e participações das entidades e seus membros em debates pela saúde pública.

Partimos da hipótese de que as agremiações não romperam integralmente com a lógica mercantilista e privatizante do modelo de saúde até então vigente a fim de elaborar novas bases políticas e sociais por meio de uma profunda reforma, uma vez que tentaram conciliar o privado no arcabouço estrutural da saúde pública. Cebes e Abrasco, durante o processo da reforma sanitária, defenderam a participação do setor privado no projeto reformista, posicionando-se contrariamente ao projeto de estatização integral da saúde apoiado por outros grupos da esquerda brasileira na área da saúde.

## Cebes e Abrasco

Em 24 de setembro de 1976, como expressão dos movimentos de luta pela democracia e transformação da saúde brasileira, nasceu o Cebes. Para Escorel (1998), a criação do Cebes representou um marco inicial de organização do movimento sanitário, e em seu percurso fundador destacam-se os papéis de militância política e de formação acadêmica desenvolvidos por alunos e professores do Departamento de Medicina Preventiva e Social da Universidade de Campinas (Unicamp) a partir do final dos anos 1960. A base teórica defendida por grande parte de seus intelectuais assentava no marxismo, especialmente, apoiada na leitura de Gramsci (Fleury, 1989; Escorel, 1998), ao mesmo tempo que dialogava com diferentes autores das ciências sociais, com a intenção de incorporar ao campo da saúde a perspectiva histórico-social.

O Cebes dividia sua linha de atuação na produção, divulgação e distribuição da revista *Saúde em Debate* e na participação em atividades acadêmicas e ações político-sociais que envolvessem a área da saúde. A agremiação reivindicava o *status* de suporte teórico e organizadora das vontades coletivas dos profissionais de saúde, das organizações da sociedade civil nas lutas do setor da saúde e pela democracia no país, conforme assinala Escorel (1998, p.88),

o Cebes é a ‘transposição’ de um pensamento nascido nas universidades para o seio da sociedade civil e faz a articulação do movimento sanitário com os demais movimentos sociais. Defende os interesses da população, sem estar diretamente vinculado a ela – ou melhor, em sua trajetória, esteve vinculado mais diretamente à academia ou à política institucional, parlamentar ou executiva.

Para Sônia Fleury (Sônia…, 2005), a ideia de que o Cebes representava “um verdadeiro partido sanitário” vincula-se também com a perspectiva defendida pelo Partido Comunista Brasileiro (PCB), uma vez que o Cebes era hegemonizado pelos militantes do partido, mas também comportava diferentes correntes e posições políticas internamente, não permitindo dizer que seria um braço do PCB. Para tanto, a política adotada pela agremiação seguia a agenda do partido, de modo que, “quando todo mundo estava querendo fazer a revolução na área da saúde, nós encaramos fazer a reforma porque essa era a perspectiva do Partido Comunista” (Sônia..., 2005).

A revista *Saúde em Debate*, editada e publicada pelo Cebes, traduz e expõe ideias, projetos e perspectivas da agremiação, e se tornou o principal instrumento de difusão dos interesses e agendas do grupo. A revista publicou seu primeiro número em 1976, lançando 19 edições até o término de 1987. Já no ano em que se institucionalizou legalmente a saúde pública na Constituição Federal, 1988, a revista editou quatro números regulares e um especial. A linha editorial refletia a agenda política da direção do Cebes, destinando-se aos leitores da área da saúde nos níveis acadêmico e profissional (Sophia, 2012).

Por seu turno, partilhando dos mesmos problemas, ideais e contexto histórico, e impulsionada pela criação do Cebes, nasceu a Abrasco, em 27 de setembro de 1979. A fundação ocorreu durante a primeira Reunião sobre Formação e Utilização de Pessoal de Nível Superior na Área da Saúde Coletiva, realizada na sede da Organização Pan-americana da Saúde, na cidade de Brasília/DF. Composta por técnicos, profissionais, alunos e professores da área da saúde coletiva, com o objetivo de “fundar uma associação que congregasse os interesses dos diferentes cursos de pós-graduação naquela área” (Lima, Santana, 2006, p.19).

Invenção brasileira, o termo Saúde Coletiva está hoje presente na agenda acadêmica e política de países da América Latina, do Caribe e da África. Trata-se, mais que tudo, de uma forma de abordar as relações entre conhecimentos, práticas e direitos referentes à qualidade de vida. Em lugar das tradicionais dicotomias – saúde pública/assistência médica, medicina curativa/medicina preventiva, e mesmo indivíduo/sociedade –, busca-se uma nova compreensão na qual a perspectiva interdisciplinar e o debate político em torno de temas como universalidade, equidade, democracia, cidadania e, mais recentemente, subjetividade emergem como questões principais. Foi em torno desses temas e do desafio de formar profissionais atentos à corrente de novas ideias sobre os problemas de saúde, alguns antigos, outros produtos de mudanças recentes nos campos biomédico, político e social, que se organizou, em 1979, a Associação Brasileira de Pós-graduação em Saúde Coletiva (Abrasco) (Lima, Santana, 2006, p.9).

A Abrasco abrange entidades de ensino, pesquisa e prestadores de serviços na área de saúde coletiva, cuja função se baseia em apoiar a formação dessas áreas, a qualificação acadêmica, profissional e o desenvolvimento de políticas públicas para saúde, educação e ciência e tecnologia em benefício da população. A Abrasco tem sua natureza essencialmente corporativa e de base acadêmica, mas com ampla margem de atuação, o que lhe permitiu desempenhar atividades de maneira abrangente na política social brasileira. Assim, além da formação de profissionais na saúde coletiva, a agremiação desenvolveu ao lado do Cebes importantes ações políticas no processo da reforma sanitária (Belisário, 2002).

## A saúde pública na ditadura, 1964-1985

A saúde pública durante a ditadura estruturava-se por meio da assistência médica previdenciária. A organização da saúde continha de um lado o Ministério da Saúde (MS), cujas funções eram executivas e normativas, voltado para o interesse coletivo, a medicina preventiva e os programas de vacinação e vigilância sanitária. De outro lado, ao contrário do MS, que de pouca força política e financeira dispunha, o Ministério da Previdência e Assistência Social abocanhava quase a totalidade dos recursos públicos e dispunha de enorme poder no aparelho de Estado, cuja principal atribuição consistiu no atendimento médico-assistencial individualizado, privilegiando ainda a medicina curativa e especializada, no lugar da atenção preventiva, ambulatorial e de interesse coletivo.

O fundo previdenciário era pago especialmente a partir da contribuição salarial dos trabalhadores. Os recursos públicos da Previdência Social eram escoados para a compra de serviços da rede privada, acarretando a ampliação indiscriminada do setor privado em detrimento do setor público, favorecendo a compra de medicamentos por meio de empresas internacionais, aumentando ainda mais o fosso de dependência da saúde pública brasileira. Portanto, formou-se um gigantesco complexo de acumulação de capital industrial de assistência médica privado.

O projeto público de saúde era liderado pela Previdência, com papel irrisório do MS, conformando uma política baseada na lucratividade da prestação de serviço. Além do deslocamento dos recursos públicos para os hospitais privados por meio da política de hospitalização e da prática médica curativa, individualizada e assistencialista (Mello, 1976). Os serviços de saúde, de modo geral, foram hegemonizados pelo setor privado, com ampla margem de diferença da oferta de serviços e recursos em relação àquela destinada ao setor público. Os recursos públicos eram canalizados para o setor privado em detrimento de uma política de fortalecimento dos estabelecimentos propriamente públicos. O setor privado dispunha de grande variedade de formas de prestação de serviços, entre elas o serviço médico liberal por meio do trabalho em consultório e clínicas, cooperativas e associativas, complexos hospitalares, empresas de medicina de grupo etc.

Nesse quadro de privatização e mercantilização da saúde nasceram inúmeras lutas pela reforma sanitária brasileira – entre elas situa-se a participação de Cebes e Abrasco. As agendas e estratégias de lutas das agremiações refletem em grande medida as próprias escolhas da esquerda partidária brasileira, tendo em vista que seus membros atuaram de forma ativa nos partidos.

As esquerdas partidárias brasileiras sofreram forte influência do movimento mais geral das ideias que afloraram nas esquerdas internacionais. Destaca-se o papel da socialdemocracia alemã, responsável por formular a ideia de que não seriam mais necessárias a tomada e a quebra do Estado a partir do processo revolucionário para alcançar o socialismo como formulado por Marx, Engels e Lênin. A transição para o socialismo se daria isenta de revoluções, conforme sinalizado pelo eurocomunismo.^1^

Em que pese os diferentes pontos de vista de seus interlocutores, em geral o eurocomunismo transposto para o contexto nacional defendia o socialismo pela via democrática parlamentar e como forma pacífica de superação do capitalismo. No plano da saúde, seu peso recaiu profundamente no processo da RSB; podemos citar como exemplo a defesa da ideia de democracia como valor universal apropriada por Cebes e Abrasco. Além das dessemelhanças entre seus interlocutores e diferenças de apropriação geográfica das ideias do eurocomunismo, podemos de forma resumida apontar elementos comuns e que impactaram diretamente o debate da esquerda brasileira, no Cebes e na Abrasco, quais sejam:

1) o socialismo como desdobramento do desenvolvimento da democracia, ou a democracia levada ao seu ‘limite extremo’, para usar os termos de Togliatti; 2) uma concepção de reforma como se uma revolução diluída no tempo e, portanto, portadora de leves e pequenos impactos, sem ruptura violenta; 3) uma determinada compreensão do Estado como agente das transformações, ao sabor da correlação de forças entre as classes e capaz, portanto, de funcionar a favor dos trabalhadores; 4) a aposta no sufrágio como meio efetivo de disputa do poder, capturável pelo jogador mais bem organizado, disposto e potente para o acúmulo de forças necessário à construção socialista; 5) a definição da luta pelo socialismo em etapas, onde antes da transição socialista deveria haver um estágio intermediário, caracterizado pela presença hegemônica dos trabalhadores no poder de Estado. E cabe ainda uma complementação: os socialistas italianos, desde Togliatti, têm sistematicamente negado a perspectiva de uma transição em dois tempos (um período prévio de luta democrática e logo após a ruptura), típica da formulação marxiana de revolução permanente. Tal negação era a própria negação do segundo ato, da ruptura (Dantas, 2017, p.56).

É importante registrar a presença volumosa de membros do Cebes e da Abrasco no PCB nos primeiros anos da reforma sanitária, como Sônia Fleury, José Gomes Temporão, Jaime Oliveira, Sérgio Arouca, David Capistrano Filho, Hésio Cordeiro, Reinaldo Guimarães, Carlos Nelson Coutinho e vários outros. A despeito das influências conjunturais e de direcionamento teórico-político que exerceram os partidos de esquerda em seus militantes vinculados ao Cebes e à Abrasco, os testemunhos dos personagens e a literatura especializada, grande parte confeccionada pelos próprios atores, sustentam não ter havido aparelhamento e defendem a existência de autonomia em relação aos partidos (Jacobina, 2016).

Ao tomar a saúde como campo revolucionário por natureza, seria necessário um esforço conjunto de teorização social da área, a fim de conduzir o caminho da luta. Para Fleury (2018), será com a criação e consolidação da saúde coletiva como área de estudos voltados para a política social e a teoria social da saúde que o objetivo foi alcançado. A convergência da medicina social com diferentes disciplinas das ciências sociais possibilitou a compreensão do fenômeno social e político da área da saúde.

Os intelectuais do Cebes e da Abrasco, em grande medida, defendiam que tais agremiações deveriam ser reconhecidas como sujeito e dirigente político e institucional do processo de hegemonia da saúde por meio da reforma sanitária. Argumentavam que a composição de tais agremiações as habilitava para “exercer a condição de dirigente em relação à transformação institucional, mas não possibilitava a necessária mudança cultural” (Fleury, 2018, p.55-56). Desse modo, caberia ao movimento sanitário, leia-se, Cebes e Abrasco, o processo de teorização e condução da luta política no campo da saúde, compreendendo toda a sociedade.

## A questão da estatização no processo da reforma sanitária

A revista *Saúde em Debate* trazia em seu editorial do segundo número, publicado em 1977,^2^ uma crítica ao exercício que a profissão médica vinha sofrendo nas últimas décadas. A questão dizia respeito ao progressivo desaparecimento do exercício do trabalho tradicional do médico, isto é, o trabalho autônomo, de consultório. Denunciava o contexto intervencionista do Estado na prestação de serviços de saúde e o forte crescimento de empresas médicas. Tal situação encapsulava os médicos em diferentes organizações empresariais privadas e até mesmo contratados pelo poder público, tornando-os trabalhadores assalariados, porém, sem os mesmos direitos conquistados por outras categorias de assalariados (Cebes, 1977a).

O editorial do Cebes se posicionou a favor do assalariamento dos médicos, mas um assalariamento com salário digno, com direitos e que fosse útil para o atendimento da população. A direção da agremiação entendeu que era preciso reorganizar a prestação de serviços de saúde em nova perspectiva, superando o apego ao exercício da função médica de tempos precedentes, colocando em seu lugar novas relações entre as equipes de trabalho, voltadas para lutar por um sistema de saúde público.

O editorial, do ponto de vista da questão estatização *versus* privatização, encaminhou seu posicionamento no sentido de que a medicina de lucro impedia a organização da assistência médica adequada à população. A luta por uma saúde “pública, institucional, sem fins lucrativos, cuja definição e rumos sejam dados pelas entidades legitimamente representativas dos usuários” (Cebes, 1977a, p.3), caberia aos assalariados.

No mesmo número, o sanitarista Carlos Gentile de Mello assina o artigo “A privatização dos hospitais governamentais, filantrópicos, universitários e de ensino”. Argumenta contra o Plano de Coordenação de Proteção e Recuperação da Saúde, aprovado em 1968, também denominado de Plano Nacional de Saúde, que propunha a entrega para a iniciativa privada de hospitais e demais unidades médico-assistenciais da administração pública. Mello (1977) denunciou os perigos da privatização e sobretudo da prática de pagamento por unidade de serviço, responsável por inúmeras distorções e malefícios ao serviço de saúde.

Ainda na mesma edição da revista, a temática é debatida de perto por Regina Maria Gittoni, que, na ocasião, cursava o mestrado em ciência política na Universidade de São Paulo (USP) e anos mais tarde se tornaria importante membra da Abrasco. O artigo intitulado “Privatizar ou estatizar?” discorre sobre a situação da saúde pública brasileira, demonstrando que o Estado não poderia ser visto como uma unidade. O aparelho de Estado comportava interesses contraditórios a partir das disputas entre ministério e Previdência, dominados por diferentes classes e frações de classe dominante. De um lado, aqueles em favor das grandes empresas, sobretudo das multinacionais, e, de outro lado, os defensores das pequenas e médias empresas. Para a autora, o Estado estava tomado por agentes e interesses do setor privado, claramente interessado na assistência médica. Portanto, chama atenção para compreender as disputas no interior do Estado, pois estatizar diz respeito à ação do Estado, ao passo que suas ações no contexto analisado estão sob controle dos interesses empresariais (Gittoni, 1977).

No mesmo ano, o editorial do número 3 da revista ressaltava que o principal obstáculo para a reforma sanitária era a “exploração das atividades ligadas à saúde com fins lucrativos” (Cebes, 1977b, p.3). A direção do Cebes posicionou-se contrária à política de saúde pública voltada para os interesses do setor privado, embora não deixasse claro que a saúde pública deveria ser elaborada integralmente pública e sem a presença do privado.

A questão que se colocava tanto pela direção do Cebes como por nomes importantes da agremiação dizia respeito à democratização da saúde. Emerson Merhy escreveu na edição de número 4 que não seria nos esquemas técnicos e racionalizados que os problemas da saúde seriam sanados. Isso porque tais caminhos seriam, acima de tudo, políticos. Merhy argumentou que o Estado se encontrava controlado por diferentes classes e frações dominantes do setor empresarial na área da saúde que negavam os interesses da população. A democratização deveria ocorrer tanto pelo rompimento com a ditadura como no acesso das camadas populares da sociedade civil ao Estado no campo da saúde – sem que isso ocorresse, não seria possível democratizar a saúde (Merhy, 1977).

O entendimento produzido pelos membros do Cebes e especialmente por suas diferentes diretorias era de que a relação entre Estado e saúde se estabelecia a partir da noção de que Estado se confundia com ditadura. Em outras palavras, a ideia de estatização confundia-se com o domínio do poder governamental da ditadura no setor saúde. Tendo em vista que, naquele momento vivido, o aparelho do Estado estava atravessado por diferentes classes e frações dos interesses empresariais, pensar em estatização seria o mesmo que pensar em mais domínio para tais setores, bem como em centralização da própria burocracia autoritária do poder público.

Tal perspectiva foi evidenciada no novo programa de trabalho do Cebes para o período de 1978 e 1979, aprovado em assembleia nacional dos delegados do Cebes. Definiram como um dos pontos programáticos que “o Estado é, portanto, a ‘estatização no Brasil’, tem penetrado em todos os setores da vida social, não para provocar socialização (no sentido de maior justiça social), mas para viabilizar um modelo de desenvolvimento capitalista” (Cebes, 1978, p.5).

Diante desse quadro e no contexto do processo de abertura democrática, realizou-se na Comissão de Saúde da Câmara dos Deputados o primeiro Simpósio Sobre Política Nacional de Saúde, entre 9 e 11 de outubro de 1979. O evento reuniu cerca de oitocentos participantes de diferentes organizações da sociedade civil, sindicatos, associações de classe, parlamentares e pontos de vista (Teixeira, Jacobina, Souza, 1980), sendo divido em três subtemas: recursos humanos; hierarquização dos serviços de saúde; e privatização e estatização dos serviços de saúde.

O evento é um marco nas discussões sobre a saúde pública brasileira, especialmente pelo fato de ter ocorrido a convite do governo em plena ditadura. A temática que nos interessa neste momento é privatização *versus* estatização, debate que ocorreu a partir da conferência do professor de economia da Unicamp Paul Singer. A mesa de debate reuniu posições díspares, tanto defensores da privatização como da estatização, uns mais moderados e outros mais categóricos. Não nos interessa aqui a defesa pela privatização por parte do grupo em favor dos interesses empresariais. Vejamos como se deu a defesa da estatização e sob quais aspectos.

Para Singer (1980), o elemento fundamental da discussão não reside entre decidir pela privatização ou estatização, mas discutir quem terá o controle dos serviços de saúde. Em sua posição, quem deveria controlar seria o consumidor. O objetivo principal seria pensar em como capacitar diretamente o usuário do serviço de saúde para que ele pudesse ter condições de estabelecer relações menos assimétricas com seu médico e com os agentes do serviço de saúde. Propôs a necessidade de modificar a relação médico/paciente a partir de um esforço para divulgar e tornar acessível aos usuários o saber e os limites do saber médico.

A partir de conhecimentos básicos e essenciais, almejava-se uma mudança de atitude dos médicos – até então, segundo Singer (1980), hierárquica e monopolista –, para que se tornasse menos arbitrária. Nesse sentido, defendeu ser indispensável a criação de instâncias de controle político, por parte dos usuários em todos os níveis dos serviços de saúde. Embora, em seu entendimento, tais soluções estivessem mais próximas do serviço estatizado, ressaltava que, sem tais alterações, “as soluções grandes, de caráter estatal *versus* privatização”, mudariam “mais a forma do que o conteúdo das coisas” (p.162).

Dentre os debatedores, destacamos a participação de Guilherme Rodrigues da Silva, professor da Faculdade de Medicina da USP e uma das figuras mais importantes da Abrasco e do Cebes. Na ocasião exercia o cargo de vice-presidente da recém-criada Abrasco, na gestão de 1979-1981, assumindo a presidência da agremiação na gestão de 1987-1989. Durante a oitava CNS, em 1986, atuou como relator geral do evento.

Em sua análise, o que se experimentava no país naquele momento era um duplo movimento, isto é, estatização e privatização ao mesmo tempo. O Estado, ao tomar para si o serviço de saúde, direcionou seus recursos políticos e econômicos para a saúde privada. Para o abrasquiano, o debate estatização *versus* privatização envolve inúmeras questões; entre elas, escolhas políticas, de modo que não bastaria estatizar, se as escolhas políticas visassem favorecer o setor privado na prestação de serviços públicos. Defendia a estatização total dos serviços de saúde, assim como Singer, compreendendo que o grande desafio estaria em criar mecanismos para que os trabalhadores obtivessem controle dos serviços por meio das suas representações autênticas (Brasil, 1980, p.165-168).

Por fim, cabe mencionar o posicionamento de Francisco Urbano de Araújo Filho, então secretário da Confederação Nacional dos Trabalhadores na Agricultura (Contag). Representando a entidade que, segundo ele, correspondia ao maior contingente de consumidores de serviço de saúde no país naquele momento, encaminhou a resolução interna apontando que “não resta a menor dúvida de que o serviço de saúde deve ser de responsabilidade direta do Estado, responsável direto pela distribuição da justiça, especialmente da justiça social” (Brasil, 1980, p.178).

A posição expressa pela Contag por meio de seu secretário evidenciou a impossibilidade de existência de qualquer projeto de saúde pública sob responsabilidade de interesse privado. Argumentava que, por mais humanizado e de boa vontade que viesse a ser o papel da iniciativa privada, sua natureza seria baseada no lucro, isto é, incompatível com os objetivos de distribuir serviços de saúde a toda a população. Defendia a Contag que somente o Estado poderia “agir com impessoalidade e isenção, sem olhar o *status* do cliente, vendo-o como uma pessoa humana, como um brasileiro, igual a todos nós outros, em que pese a desigualdade econômica, financeira, intelectual ou outras similares” (Brasil, 1980, p.178). Assim como Guilherme Rodrigues da Silva, entendia que o Estado vinha privilegiando o setor privado, de modo que somente a real estatização poderia dar conta dos reais problemas dos serviços de saúde.

O encontro na Câmara dos Deputados também marcou um momento histórico para a trajetória da reforma sanitária, quando Sérgio Arouca, representando o Cebes, leu o documento “A questão democrática da saúde” (Cebes, 1980), propondo pela primeira vez a criação do Sistema Único de Saúde (SUS) (Paim, 2008). Escrito por Hésio Cordeiro, José Luiz Fiori e Reinaldo Guimarães, o documento foi debatido e aprovado pelos membros do Cebes. De acordo com Paim, o documento fora um marco na construção da reforma sanitária, porque não se tratava mais de uma ideia, mas de um conjunto de proposições (Paim, 2008).

A proposta do Cebes sintetizava as principais reivindicações dos diversos coletivos que participavam do movimento sanitário. Propôs uma participação social no lugar do autoritarismo, “no lugar de políticas de controle das doenças, notadamente transmissíveis, a promoção da saúde e melhoria da qualidade geral de vida; no lugar de um setor dividido entre saúde pública e medicina previdenciária, um sistema unificado e universal” (Paiva, Teixeira, 2014, p.22).

Para o Cebes, a democratização da sociedade e a socialização da política possibilitariam a abertura de canais para a participação de amplos setores da sociedade. Assim, tornariam o aparelho de Estado permeável aos interesses populares e contrabalançariam as relações de força. Nesse sentido, o controle centralizado daria lugar à descentralização do poder, abarcando uma reforma institucional na saúde envolvendo estados e municípios na elaboração de políticas públicas para atender a suas necessidades próprias. Outra medida baseava-se em “incorporar o Legislativo em todos os seus níveis enquanto representação política da sociedade no aparelho do Estado” (Cebes, 1985, p.11). Em síntese, a formulação do projeto de descentralização compreende, segundo o Cebes,

toda a história autoritária do país (uma história autoritária que teve sua demarcação máxima com o Regime de 64, mas que faz parte da tradição e da história republicana no país) tem a tendência centralizante, a tendência da concentração de poder no âmbito federal; creio que a construção e a aplicação de uma reforma sanitária devem ter um forte conteúdo de descentralização, de democratização e participação de todos os segmentos da população. Deve ser altamente descentralizado, certamente com base nas experiências das Ações Integradas de Saúde, na reformulação e na renovação dessas experiências. E deve ter como base a consideração da extrema heterogeneidade da organização do sistema de saúde nas várias regiões do país, reverter o modelo concentracionista que privilegiou as regiões Sul e Sudeste, privilegiou em termos de distribuição de recursos, de equipamentos e de recursos humanos. Significa considerar essa heterogeneidade, significa, talvez, construir múltiplos sistemas unificados de saúde com base estadual e regional, sob a orientação de um órgão ou de órgãos centrais que sejam formuladores e avaliadores de uma política de saúde, mas que, realmente, a execução, a programação, o ajuste se dê no âmbito específico das realidades regionais estaduais, como forma de reversão desse modelo concentracionista, desse modelo perverso e desigual que discrimina de uma forma tão patente os vários segmentos da população urbana e rural, Norte e Nordeste, Sul e Sudeste, populações dispersas, e assim por diante (Cebes, 1985, p.11).

A leitura do documento feita por Sérgio Arouca sinalizava que o sistema de saúde deveria ser descentralizado, organizado de forma política e administrativa em todos os níveis (federal, estadual e municipal). Portanto, a descentralização tinha por objetivo

viabilizar uma autêntica participação democrática da população nos diferentes níveis e instâncias do sistema, propondo e controlando as ações planificadas de suas organizações de partidos políticos representados no Governo, assembleias e instâncias próprias do sistema único de saúde. Este talvez seja o ponto fundamental dessa proposta, negador de uma solução meramente administrativa ou ‘estatizante’. Trata-se de canalizar as reivindicações e proposições dos beneficiários, transformando-os em voz e voto em todas as instâncias. Evita-se também, com isto, uma participação do tipo centralizador, tão cara ao espírito corporativista e tão apta às manipulações cooptativas de um Estado fortemente centralizado e autoritário, como tem sido tradicionalmente o Estado brasileiro. Estabeleça o estatuto de convivência entre a prática assalariada vinculada ao sistema único de saúde e a autêntica prática da medicina de consultórios particulares, que têm tradição na medicina brasileira (Brasil, 1980, p.229).

Um fator em comum dos editoriais do Cebes e dos trabalhos individuais de seus membros é de que não adiantava mudar os planos e projetos para o sistema de saúde nacional, pois os agentes permaneciam com os mesmos interesses empresariais e mantinham a mesma organização privatizante e mercantilista. A proposta do Cebes que vigorou ao longo do processo da reforma sanitária, em grande medida, pode ser vista como uma resposta aos problemas levantados pelos debatedores supracitados na mesa sobre privatização *versus* estatização. Ao descentralizar e criar mecanismos de controle dos usuários nos serviços de saúde, possibilitaria segundo tal proposição a democratização da saúde. No entanto, a democratização para sair do discurso e se tornar real não ocorreria pela simples mudança governamental acerca dos planos e projetos políticos para o sistema de saúde nacional.

Cebes e Abrasco foram implacáveis na crítica ao modelo centralizador e intervencionista do Estado ditatorial brasileiro. O quadro da saúde pública sob comando do autoritarismo era denunciado pelas agremiações como de um completo “caos” na organização da assistência em saúde. Apontavam a convivência de diferentes modalidades assistenciais, diferentes formas de tratamento, de atendimento e valores cobrados (Cebes, 1985).

Diante do caos causado pela política de saúde pública e a presença hegemônica do setor privado, as agremiações entendiam que não existiam “condições, quer econômicas, quer políticas quer técnicas, para o contexto de um regime de transição democrática, prescindir-se da iniciativa privada, responsabilizando-a simplesmente pelos problemas de nossa assistência à saúde” (Cebes, 1985, p.10). Como alternativa viável para tais problemas, propuseram a construção de um Estado mediador e regulador entre os interesses privados e da população, possibilitando a presença dos diversos segmentos sociais na elaboração de políticas públicas de saúde.

## Público *versus* privado no contexto da redemocratização

Eleutério Rodriguez Neto (1985), ex-presidente do Cebes, no artigo “Subsídios para a definição de uma política de atenção à saúde para um governo de transição democrática”, no número 17 da revista *Saúde em Debate*, em 1985, defendeu a proposta racionalizadora para o projeto de saúde pública. Tal proposta implicava, entre outros aspectos, a responsabilidade do Estado sobre o sistema de saúde, tendo o setor privado como complementar e subordinado.

A descentralização era o elemento fundamental da proposta, e, para não ser confundida com um processo de “estadualização” ou “municipalização”, Rodriguez Neto (1985) esclarecia que a descentralização, embora devesse estar contida em seus aspectos, ultrapassaria os limites estaduais e municipais para se configurar como a “desconcentração do poder entre os vários níveis, até o de ‘ponta de linha’” (p.15). A descentralização nessa concepção referia-se a instâncias gestoras do sistema em todos os seus níveis, tendo poder decisório. No entanto, mantendo um caráter multi-institucional, isto é, com participação do governo federal “como forma de assegurar a equanimidade de critérios técnicos financeiros e políticos no avanço desse processo de democratização” (p.15).

Ainda segundo o sanitarista, devia se construir um novo “pacto” entre o setor público e o privado, a fim de superar a relação existente no contexto ditatorial. O pacto previa: qualificar o setor público como modelo e padrão de eficiência e eficácia; a participação dos prestadores de serviços nas discussões sobre as remunerações dos seus serviços; regionalizar e hierarquizar os serviços públicos e privados numa mesma rede; redimensionar os serviços contratados, tendo em vista as reais necessidades de cobertura e as prioridades do setor público em cada região; descentralizar o controle da qualidade assistencial. No novo pacto o Estado não devia subsidiar ou tutelar o setor privado, deixando-o à livre iniciativa (Rodriguez Neto, 1985, p.16).

Na mesma linha, o Conselho Nacional de Secretários de Saúde (Conass), no documento “A questão da saúde no Brasil e diretrizes de um programa para um governo democrático”, defendeu o estabelecimento de um sistema único de saúde, descentralizado e universal. Do ponto de vista do setor privado, apontou que o setor público deveria normatizar e regular as ações de saúde, de modo que o privado deveria “desempenhar, no Sistema Unificado de Saúde, um papel suplementar ao do setor público, especialmente na rede hospitalar onde tem uma presença significativa” (Conass, 1985, p.21-22).

Tais proposições se juntaram a tantas outras de diferentes entidades e parlamentares durante o quinto Simpósio de Política Nacional de Saúde, realizado pela Câmara dos Deputados, em novembro de 1984. O relatório final serviu de parâmetro para o governo de transição, contendo como sugestão a presença do setor privado como suplementar na saúde pública. Nesse contexto de transição e disputas de projetos para a saúde brasileira, ocorreu a oitava CNS, em Brasília, em 1986.

A oitava CNS marcou um dos principais momentos da reforma sanitária, considerada pela literatura especializada como momento central para a formatação do projeto sanitário. Representou também a primeira conferência a permitir a participação dos usuários e da sociedade civil organizada da saúde nos debates.

Um dos temas de maior divergência ao longo da conferência foi a estatização integral *versus* a estatização progressiva da saúde. O Partido dos Trabalhadores e o Partido Democrático Trabalhista defenderam a “estatização já”, enquanto o PCB e o Partido Comunista do Brasil, o Cebes e a Abrasco, entre outros, defenderam a estatização progressiva por meio da estratégia de conciliação com o setor privado (Rodriguez Neto, 2019, p.91).

Logo na abertura da conferência, o presidente da comissão organizadora, Sérgio Arouca (1987), proferiu em seu discurso que o setor privado, mesmo ausente do evento, seria representado e defendido em seus interesses por aqueles que estavam presentes. Arouca deixou claro em seu pronunciamento a proposta de não conflitualidade com o setor privado na confecção do projeto da reforma sanitária, assim como estabeleceu como princípio a noção de que o projeto de saúde pública não seria elaborado sem a presença do setor empresarial, isto é, confirmou a posição de Cebes e Abrasco de complementariedade do privado no projeto de saúde pública, no lugar da defesa pela estatização integral da saúde. Em suas palavras,

Há uns dias atrás, algumas entidades ligadas ao setor privado se retiraram da Conferência, alegando que, como representavam uma grande percentagem dos serviços de saúde prestados ao País, deviam ter maior número de delegados. Mas se equivocaram. No meu entender, essa proporção de serviços prestados não corresponde à proporção da população brasileira. E esta é uma Conferência da população brasileira e não uma Conferência dos prestadores de serviços. Mas lamento profundamente a sua ausência, porque nesta Conferência está se tratando é de criar um projeto nacional que não pretende excluir nenhum dos grupos envolvidos na construção da saúde do povo brasileiro. Assim, a eles queria deixar uma mensagem: que, mesmo na sua ausência, vamos estar defendendo os seus interesses, desde que estes não sejam os interesses da mercantilização da saúde. Portanto, todo aquele empresário que está trabalhando seriamente na área da saúde, na qualidade da sua competência técnica e profissional, não precisa se sentir atemorizado, porque aqui ele vai ser defendido (Arouca, 1987, p.39).

O posicionamento de Arouca foi acompanhado de perto por seus colegas ao longo da conferência. Jairnilson Paim questionou em que medida a estatização da saúde serviria para atender aos interesses da população. Em sua avaliação, o que vinha ocorrendo na política estatal de saúde era o contrário, uma vez que a organização da saúde favorecia os interesses privados. Levantou como questão a pertinência em manter essa política de privilegiamento do setor privado, isto é, “um setor privado incapaz de se manter no mercado sem a proteção paternalista do Estado” (Paim, 1987, p.56).

Paim questionava ainda se os serviços de saúde deviam ser do poder público e se a saúde poderia ser entendida como um serviço público. As dúvidas levantadas por Paim (1987) são interessantes, pois ele colocava no centro do debate a dificuldade do estabelecimento da saúde democrática, universal e acessível a todos. Em sua análise, a trajetória brasileira deixava claro que público não significava estatal. A política e os serviços estatais de saúde, em vez de ser públicos, demonstraram ao longo dos anos sua natureza privatista. Sinalizava como alternativa a participação dos cidadãos na política de saúde por meio do controle público dos serviços estatais e da gestão das instituições.

Nesse sentido, uma das grandes questões enfrentadas pelos participantes da oitava CNS e por todo o percurso da reforma sanitária até a promulgação da Constituição de 1988 correspondeu a saber se a transformação da política de saúde implicaria sua estatização.

Para a vice-presidenta da Abrasco na época, Sônia Fleury, a resposta seria “não necessariamente”, pois não bastaria estatizar a saúde, sendo o Estado o formulador da política nacional de saúde, mas ao mesmo tempo manter o modelo centralizador, autoritário e favorecer o setor privado como historicamente tem sido feito. Entendia o Estado como centralizador e autoritário, sobretudo após 1964, quando ocorreu a modernização administrativa e institucional dos aparelhos de Estado e seus organismos administrativos, gerando um maior nível de especialização de cada órgão e uma crescente centralização e concentração de recursos (Fleury, 1987, p.107).

Fleury (1987, p.120) defendeu durante a oitava CNS que o controle financeiro do Estado no novo pacto social seria necessário, porém, insuficiente, “tornando-se imprescindíveis redefinições de nível jurídico desta nova relação”. Hésio Cordeiro, presidente da Abrasco entre 1983 e 1985, complementou afirmando que a relação público e privado deveria ser reformulada, para evitar desvios de toda ordem, de modo que não poderia se dar a partir do campo do direito civil; ou seja, a reforma sanitária exigiria um novo marco no relacionamento jurídico entre o público e o privado, estabelecendo o contrato jurídico-padrão assente no direito público. Assim, o setor privado prestaria serviço para o setor público, tendo como fundamental o compromisso com o interesse público e da coletividade, firmado em contrato de direito público, e não de direito civil (Cordeiro, 1987, p.148).

Já existem experiências na sociedade brasileira em que serviços essenciais, como os transportes e telecomunicações, são monopólios estatais, sendo a prestação de serviços realizada por empresas privadas, consideradas, como concessão. Neste caso, o serviço é assumido como um bem público essencial, permitindo ao Estado mecanismos legais de controle e intervenção sobre os prestadores privados. É necessário avaliar a aplicabilidade dessas experiências ao setor saúde (Fleury, 1987, p.110).

A relação entre público e privado prevista por Fleury seria possível a partir da existência de um Estado democrático capaz de socializar a política, permitindo o controle social efetivo de forma descentralizada e desconcentrada. Em sua concepção, a estatização estaria ligada diretamente à tecnoburocracia estatal garantidora da acumulação de capital.

Seguindo a mesma linha interpretativa, Cristina Possas, então pesquisadora da Fiocruz e integrante do Cebes e da Abrasco, defendeu uma reformulação na relação jurídica e institucional entre os setores público e privado. Apostou na descentralização da saúde, operada, entre outros fatores, por meio do regime de concessão ao setor privado, que atuaria de forma complementar no sistema de saúde. Para Possas (1987, p.247), o modelo de concessão então implementado em outros setores, como telecomunicações e transportes, “propiciaria ao Estado maior controle e a possibilidade de intervenção sobre a compra dos serviços privados em saúde”.

Isso ocorreria em concomitância com a criação de instâncias de participação popular e dos trabalhadores no sistema de saúde público. Tais fatores conjugados seriam capazes de “romper com as atuais distorções no relacionamento com o setor privado, garantindo sua subordinação ao interesse público” (Possas, 1987, p.247). Para a autora, não se tratava “de retomar o já desgastado debate da estatização *versus* privatização do sistema de saúde, mas sim de garantir ao menor custo social novas formas de relacionamento entre os setores público e privado, marcadas pela transparência e submetidas a um planejamento democrático” (p.250).

Na concepção de Eleutério Rodriguez Neto, a proposta apresentada de reformulação da saúde e criação do Sistema Único de Saúde não teria por intenção a estatização ou eliminação da iniciativa privada na saúde:

As normas vigentes no relacionamento necessário do poder público com o setor privado deverão ser subordinadas aos requisitos técnicos e financeiros para uma cobertura assistencial universal e igualitária e cujas condições podem ou não ser aceitas pelo contratante para efeito de participação ou não no sistema público de prestação de serviços de saúde. Não se trata aqui da livre iniciativa, financiada diretamente pelo recurso particular, individual ou cooperativo (seguro), que, não ferindo os preceitos éticos, pode organizar-se livre e independentemente da tutela do Estado (Rodriguez Neto, 1987, p.263).

Na mesma direção, a Confederação Nacional das Classes Trabalhadoras, representada por Luís Roberto de Oliveira, defendeu durante o evento a criação do SUS, sob comando único e dever do Estado. Propuseram que o sistema de saúde “prestigie de maneira predominante o setor público e o setor privado não lucrativo representado pelas Santas Casas, pelos hospitais beneficentes e que o setor lucrativo entre de maneira complementar e subordinado ao controle oficial, ao controle do Estado” (Brasil, 1987, p.229).

Por outro lado, o representante da Central Única dos Trabalhadores (CUT) na oitava CNS, Arlindo Chinaglia Júnior, rebateu os argumentos de Fleury, apontando que o debate sobre a saúde não podia prescindir da relação de poder na sociedade. Em nome da CUT, afirmou que as transformações na sociedade e na saúde só se efetivaram de fato a partir do controle dos trabalhadores, “mas isso não está colocado no momento. No momento, na verdade, faz-se um discurso, e, na prática, faz-se uma aliança de classes, inclusive com a classe dominante” (Brasil, 1987, p.120). Acrescentou ainda como questão a se resolver, “sob a égide de que estatização pode ser autoritária podemos assistir à abertura da porta, escancaradamente, apesar do discurso, à iniciativa privada” (p.120).

A CUT respondeu a Fleury afirmando que eram favoráveis à estatização da saúde, realizada a partir do controle dos trabalhadores, como forma de romper com o autoritarismo. Por fim, conclui conclamando os participantes a defender a estatização, considerando que a estratégia não podia se colocar no plano da negociação no nível do poder de Estado, uma vez que a estratégia deveria ser pautada pela organização, a conscientização e a luta da classe trabalhadora (Brasil, 1987, p.120).

Por fim, na apresentação dos relatórios das pré-conferências estaduais, apenas o estado de Goiás apresentou proposta alternativa à participação complementar do setor privado no sistema de saúde público. Aprovaram a “estatização gradativa da rede hospitalar e ambulatorial, responsável pelo atendimento da população, definindo as instituições privadas temporariamente, as funções complementares do sistema” (Brasil, 1987, p.358).

## Considerações finais

Cebes e Abrasco, ao longo do processo da reforma sanitária até 1988, adotaram a estratégia de conquistas de direitos de cidadania para a área da saúde travando as lutas por dentro do aparelho de Estado. Para Dantas (2020), ao apostar na democratização como valor universal, baseada, entre outros fatores, na retomada os pleitos eleitorais, no controle democrático do Estado, na socialização da política e, consequentemente, da organização de uma sociedade civil progressista, unindo diferentes classes e frações de classe em prol de um projeto de saúde pública, “se não produziu uma subestimação das forças representantes do capital, no mínimo relativizou o seu papel de classe e supervalorizou o poder de fato de uma luta política de esquerda crescente e deliberadamente canalizada para o interior da máquina do Estado” (p.168).

Em sua análise, para além dos argumentos do movimento sanitário (especialmente, Cebes e Abrasco) contrários à estatização da saúde, as motivações pareciam ser outras; estavam alicerçadas em suas estratégias de

convergências multiclassistas em defesa da democracia – democracia esta assumida pelas forças majoritárias de esquerda, então, como um ‘valor universal’, isto é, uma fronteira que não deveria ser ultrapassada, um limite de respeito à ordem e às regras do jogo democrático que expressaria um compromisso entre as classes pelo não golpe (burguesia) e por um caminho pacífico para o socialismo (trabalhadores) (Dantas, 2020, p.169).

A estratégia democrática do grupo formado por intelectuais de Cebes e Abrasco certamente responde a grande parte das motivações para a negação da estatização da saúde brasileira. A conciliação de classes consequentemente conduziu à não identificação do setor privado como inimigo de classe no projeto político do grupo. A democratização da saúde se colocou acima das classes; no entanto, talvez tivessem superestimado mais a si próprios do que subestimado as forças do capital. O setor privado não foi alçado como inimigo de classe, como antagônico ao projeto da reforma sanitária; o projeto se baseou menos em se contrapor ao seu inimigo e mais nos seus fins, ou seja, a saúde de natureza democrática e universal.

Os problemas da saúde seriam resolvidos com a existência de um sistema público, capaz de atender de forma universal, igualitária e democrática à saúde a todos, isto é, o Estado deveria prover e promover a saúde pública a toda a população, por sua vez, não mexeria no setor privado e na livre iniciativa na área da saúde. Alteraria, por outro lado, a forma como a relação entre público e privado se estabeleceu no período ditatorial de amplo deslocamento dos recursos e poder para o privado. Nesse sentido, no limite do projeto reformista, supõem-se que cada um poderia ter sua fatia da área da saúde em livre concorrência. No entanto, em vez de haver dois polos diferentes e antagônicos de saúde, propuseram e defenderam a presença do setor privado na saúde pública por meio de contrato de direito público e convênios, atuando de forma a complementar os serviços de natureza pública.

Ora, o argumento de que não havia condições conjunturais para a estatização total da saúde por conta da presença e do poder significativo, diríamos, hegemônico do setor privado no setor na saúde, conduziu a estratégia da “dialética do possível”. Se não é possível estatizar, que fique o privado com sua livre iniciativa, e o setor público com a saúde pública e universal. Porém, essa não foi a proposta do grupo, pois acreditava que o novo modelo de saúde que sairia expresso na Constituição federal não poderia prescindir da atuação do setor privado na execução de serviços públicos, devido a seu atraso nas estruturas física, material, de atendimento etc.

O sistema público de saúde, portanto, já nascia com o setor privado em sua institucionalidade. Para as agremiações, o privado não era o problema para a saúde pública no projeto da reforma sanitária; o problema era a forma como a saúde pública relacionava-se com o privado. A conquista do espaço empresarial no projeto de saúde pública não resultou de embates com o setor privado, e, sim, da opção pela não conflitualidade e harmonia entre opostos. Cebes e Abrasco defenderam o setor privado, embora não fossem seus representantes.

## NOTAS

1 Em linhas gerais, o eurocomunismo refere-se aos partidos comunistas, especialmente de Itália, França e Espanha, que visavam estabelecer uma perspectiva diferente do comunismo da URSS e propor alternativas para o socialismo internacional a partir do ponto de vista europeu ocidental e da defesa do socialismo democrático.

2 O conselho editorial da revista era composto por Ana Maria Segall Corrêa, Dalmo Herrera Feitoza, David Capistrano Filho, Emerson Elias Merhy, José Ruben F. Alcântara e Sandra Roncali Mafezolli.
